# Intellectual Disability and Dental Services: Experience from Israel

**DOI:** 10.3389/fpubh.2014.00133

**Published:** 2014-09-30

**Authors:** Ilan Feldberg, Joav Merrick

**Affiliations:** ^1^Division for Intellectual and Developmental Disabilities, Ministry of Social Affairs and Social Services, Jerusalem, Israel

**Keywords:** intellectual disability, dental services, Israel, dental clinics, dental survey

## Introduction

People with intellectual and developmental disability (IDD) have often been neglected by the dental profession, because of a variety of barriers, like insufficient professional knowledge and experience to treat this population, lack of cooperation by the person with IDD, lack of awareness by carers, inadequate facilities, and inadequate compensation for treating this population, who takes longer time to approach, assess, and treat (1, 2).

In general, this population has poor oral health and oral hygiene. Data indicate that people with IDD have more untreated caries, higher prevalence of gingivitis, and other periodontal diseases affecting their ability to chew, speak, and look attractive (3). With increasing age and life expectancy this population is in need of good dental care on a regular basis to prevent disease and improve quality of life (4, 5).

In another publication (6), we looked at a systematic review of 27 original papers (7) that showed this population has poorer oral hygiene, higher prevalence, and severity of periodontal disease. Concerning the rate of caries in this population, it was observed the same as or higher than the general population, but untreated caries was found constantly higher. Down syndrome and people with IDD unable to cooperate with routine dental care were at high risk for oral health problems (6, 7).

## Dental Care in Israel for this Population

In this paper, we would like to present the experience of a national dental service for people with IDD and show the trends that have lead to a unique service in our opinion. In Israel, the Division for Intellectual and Developmental Disabilities of the Ministry of Social Affairs and Social Services has the responsibility for the assessment, treatment, rehabilitation, and service for persons with IDD. Out of a total population of about eight million, the Division is in contact with about 35,000 persons of all ages with intellectual disability (8). Dental services are provided free on a national basis by the Health Services of the Division to all persons in residential care (about 10,000 persons) and optional to persons with IDD living at home.

We have conducted several surveys over time in order to monitor the dental health status of people with IDD (6) and we will here repeat some of the findings. The first dental survey was conducted in 1971–1972 by the School of Dental Medicine at Tel Aviv University at three government residential care center, including 573 residents with IDD out of a total residential care population of 2,700 (6, 9). During this period in time, there were no dental clinics available for this population and the study was conducted via a mobile dental clinic (a bus) with four dental chairs (6, 9). It was found that only 20% of the residents were able to fully cooperate and 84% of all residents were in need of intervention. About 50% of the residents needed pre-medication or general anesthesia (GA) with over 70% suffering from caries in various degrees and 25% showed serious damage (seven and more decayed teeth per person). About 60% had lost one or more teeth and 17% more than seven teeth, whereas only 30% had fillings in one or more teeth (6, 9). The survey found 2,281 decayed teeth with a male to female ratio of 0.58. As a result of this study, the Ministry decided to establish dental clinics in residential care centers for this population. Today 20 clinics provide service to the whole population in residential care and in recent years also people with IDD living at home, persons with autism, and other severely disabled persons in residential care centers in Israel (6, 10).

In 1993, a second survey with a randomly selected sample was conducted (6, 11) among 462 persons with IDD in residential care. Three hundred sixty-two were able to collaborate with the study and in the 2- to 11-year-old group decayed teeth was found at *D* = 12.84, no missing teeth and only 1.89 filled teeth making decayed/missing/filled teeth (DMFT) of 13.93. In the 12- to 14-year age group, an active caries level of 12.15% was found, no missing teeth, filled teeth at 0.73% and DMFT at 12.88. Caries level in 15- to 24-year-olds at 14.18% and DMFT at 17.96, while 25- to 34-year-olds showed a DMFT at 21.07 and the 35 years and older an increased DMFT at 18.52 (6, 11).

In 1995, we conducted a third large scale study together with the Hebrew University Hadassah School of Dental Medicine in Jerusalem (6, 12) with a random sample of 387 persons. In this study, we divided the subjects into four functional groups according to their level of intellectual disability: (1) educable (*n* = 70), (2) trainable (*n* = 92), (3) behavioral problems (*n* = 106), and (4) severe physical impairment (*n* = 119). Total age-adjusted DMFT was 12.78, which differed significantly by behavioral group (*p* < 0.001); the MT was 10.70 for the educable group compared with 5.52 for the group with severe physical impairment. This study found a total treatment need per participant with a mean of 3.32 for restorations and 0.61 for extractions. Residential care centers with a dental clinic had higher participant mean DMFT, DT, MT, and FT values (*p* < 0.05) compared with those institutions which had no clinics (16.04 vs. 9.74; 5.17 vs. 5.06; 9.45 vs. 4.16; 1.41 vs. 0.52). The age-adjusted community periodontal index of treatment needs (CPITN) scores showed significantly difference by behavioral group; the group with IDD and severe physical impairment had the highest CPITN 3 category with a mean score of 2.93 compared with *x* = 1.89 for the educable group; however, the educable group had the most sextants with no teeth (*x* = 2.48). This study confirmed high dental morbidity and significant oral health differences, when looking at behavior group, age and dental clinic status (6, 12).

The last survey (13) during the years 2007–2012 was based on statistical data on the general Israeli population dental condition compared to a sample of 6,000 persons with IDD in residential care and 5,400 persons with IDD from special education schools, sheltered workshops, and day centers in Israel. Like the previous surveys, we found the dental condition of people with IDD considerably worse than the general population and the dental condition of residents better than the dental condition of people with IDD living in the community.

Both subgroups (residents and non-residents) face common conditions and difficulties, but the residents group has advantages, like dietary control, free and accessible dental care, and the special dental system of the Health Service. The main components of this special dental service are: accessibility, constant, and well-trained dental staff, salaries based on a global base versus fee for service, professional use of behavioral, and protective immobilization techniques to reduce the need for GA, long term maintenance, and surveillance.

We have also conducted several community studies (6), but with smaller number of participants or with specialized populations like, for example, Down syndrome (14, 15). In one general study of aging and intellectual disability (10) from the Jerusalem area, we compared persons (40 years and older) living in community residence (*n* = 65) with those living at home (*n* = 43). We found that health problem had already developed by age 40 years with visual (33%) and hearing impairments (20%) or dental problems (30%). People with IDD in community residence group displayed more medical problems, while individuals living at home had more dental problems. This particular study resulted in the Ministry agreeing to open up residential care dental clinics for people with IDD living at home (6, 10). The dental findings are not unique to Israel, but also found in other countries like the United States (16), United Kingdom (17), and India (18).

## Twenty Dental Clinics

Today, we have 20 dental clinics to service this population with clinics located on the premises of residential care centers (6). Each dental clinic is usually in close connection with the general medical clinic of the residential care center with a waiting room area to facilitate people from other centers or the community waiting for dental care (6). We have tried to have two dental chairs in each clinic in order for the dentist and the dental hygienist to work at the same time on several patients or in some cases two dentists working at the same time. We have tried to create a friendly environment in the clinics, but we are still working on improvements and have introduced the snoezelen or controlled multisensory stimulation concept in some of our clinics (6, 19). In order to facilitate people in wheelchairs, the dental chairs are specially designed and the clinic also equipped with lift and papoose board as standard equipment (6).

We have found that it is important to have a constant, experienced, and dedicated staff in order to provide good treatment for this population. We also train the staff to show respect and understanding for patient. If the patient will see the same dentist, dental assistant, and dental hygienist again and again, it will be less anxiety and rather a pleasure to come for the minimum of a yearly visit in the dental clinic (6).

At the first visit, we have established a standard assessment procedure and since 2002, we have implemented a national computerized recording system (6). We have also implemented a behavioral technique of tell-show-do (TSD), where the persons with IDD are told what the operator is going to do in non-threatening terms (6). We have also conducted frequent educational programs and training for the staff to improve service (6).

Another unique instrument in the routine dental care is the papoose board (medical restraint device), a device commonly used to immobilize a person for dental work, blood-drawing, and minor medical procedures (6). The papoose board is standard equipment in each dental clinic and we feel that this procedure is preferable and less risky to sedation or GA (6).

We have also in recent years introduced nitrous oxide or laughing gas in most of our clinics (6).

We prefer to use the papoose board with as many patients as possible and in our last published annual survey of residential care centers (20), we found that out of 6,988 residents only 38 (0.54%) needed GA. This is a drastic decrease since our first annual survey in 1998 (21), where 266 out of 6,022 residents (4.42%) needed GA. We therefore recommend papoose board as standard equipment for all dental clinics working with this population.

## Conclusion

In this short paper, we have shown that people with disabilities, although suffering from the same diseases like the general population, need special medical and dental systems with professional expertise to face those special needs. The treatment might be the same, but more time consuming and needs expertise in communication matched to each individual.

Prevention in medicine and dentistry, which is the best and the cheapest way of providing good health long term cannot be done and learned without communication, understanding, and cooperation, again emphasizing the need for special medical/dental systems.

In Israel, our experience has taught us to recommend a yearly routine visit to a dental clinic for all persons with intellectual disability at all ages from the eruption of the first tooth. It is important to us with this recommendation and even concerning people without teeth in order to assure a healthy oral mucosa. Medical problems and dental problems often interrelate and we therefore recommend a close collaboration with the treating physician. In recent years, we have also implemented a major program of dental implants to further improve the dental status of this population.

We would like to see further research in the dental care of this population, further education and specialization of dentists and dental hygienists in this field of service for people with intellectual and developmental disabilities.

## Author Note

Part of this text also figures in an earlier publication with permission (6).

## Conflict of Interest Statement

The authors declare that the research was conducted in the absence of any commercial or financial relationships that could be construed as a potential conflict of interest.
